# Effect of Biochar on Soil Properties, Soil Loss, and Cocoyam Yield on a Tropical Sandy Loam Alfisol

**DOI:** 10.1155/2020/9391630

**Published:** 2020-02-25

**Authors:** Aruna Olasekan Adekiya, Taiwo Michael Agbede, Adeniyi Olayanju, Wutem Sunny Ejue, Timothy A. Adekanye, Titilayo Tolulope Adenusi, Jerry Femi Ayeni

**Affiliations:** ^1^College of Agricultural Sciences, Landmark University, P.M.B. 1001, Omu-Aran, Kwara, Nigeria; ^2^Department of Crop, Soil and Pest Management Technology, Rufus Giwa Polytechnic, P.M.B. 1019, Owo, Ondo, Nigeria; ^3^Department of Agricultural & Biosystem Engineering, Landmark University, P.M.B. 1001, Omu-Aran, Kwara, Nigeria

## Abstract

Among agricultural soil amendment that can enhance crop productivity and soil sustainability is biochar. Hence, two-year field experiments were conducted on a sandy loam Alfisol at Owo, southwest Nigeria, to evaluate the effects of biochar produced from hardwood on soil physical and chemical characteristics, erosion potential, and cocoyam (*Xanthosoma sagittifolium* (L.) Schott) yield. The study was a 2 × 4 factorial experiment with two years (2017 and 2018) and four biochar levels (0 (control), 10, 20, and 30 t ha^−1^). The treatments were laid out in a randomized complete block design with three replications. Results indicated that biochar application significantly in both years improved yield of cocoyam and soil physical (bulk density, porosity, moisture content, mean weight diameter (MWD) of soil aggregates, dispersion ratio, and infiltration rate) and chemical (soil organic matter, pH, N, P, K, Ca, Mg, and CEC) properties and erosion resistance. Soil characteristics and cocoyam yield improved with level of biochar from 0–30 t ha^−1^. When 2018 is compared with 2017 in term of soil loss, in the amended plots, 2018 reduced soil loss by 7.4, 20, and 73.5%, respectively, for 10, 20, and 30 t ha^−1^biochar, whereas there was an increase of 2.7% soil loss in the control plot in 2018 compared with 2017. Therefore, application rate of 30 t ha^−1^ biochar is considered as suitable for severely degraded soil because this application rate efficiently improves cocoyam yield and soil properties and reduces soil loss.

## 1. Introduction

One of the major constraints to crop production in the tropics is soil-related problems. In Nigeria, the largest soil order—Alfisol—is faced with a lot of unfavourable challenges such as low fertility, soil acidity, weak structure and high susceptibility to crusting, compaction, and accelerated erosion [1]. Also, the expansion of agriculture into marginal areas, deforestations, the shortening or elimination of fallows, inappropriate farming practices, and low input inevitably have several environmental and economic impacts on tropical soils where the resilience ability of the soil is limited [2]. Therefore, the avoidance of soil loss by improved management of the natural resources is important to combat low agricultural production, food insecurity, and the increase in level of poverty in tropical countries [3].

Among agricultural soil amendment that can enhance agricultural productivity and soil sustainability is biochar. Biochar is the product of pyrolysis of organic materials in the absence of oxygen and at high temperature [4]. Until now, uncharred amendments were being used to improve the fertility/productivity of the soil and improve organic matter content [5]. However, the decomposition of soil organic matter is too high [6] especially under tropical condition with high temperature, and therefore biochar provides an additional soil management option. Due to its relative recalcitrant after amendment, biochar can remain in the soil for many years. This contrasts with crop residues or animal manures which turn over on a decadal timescale [7].

Soil physical and chemical characteristics have a direct effect on soil productivity for crop production [8]. Studies have showed that biochar application improved the physical, chemical, and biological properties and therefore crop yield. Biochar has been shown to improve soil structure [6], soil aggregate stability and porosity [9, 10], water-holding capacity and nutrient cycling [11, 12], tensile strength and penetration resistance [13], and soil infiltration and reduce runoff and decrease erosion [14]. It is also more stable than any other soil amendment and increases nutrient availability beyond a fertilizer effect [15]. Research results have also shown that biochar can ameliorate soil nutrient status, cation exchange capacity, nutrient use efficiency, and nutrient holding capacity and decrease soil acidity [4, 16, 17].

Despite the results from previous studies on biochar, there are still scanty literature and data on the use of biochar for improving soil physical and chemical characteristics and crop yield and reducing soil loss especially on Alfisol of southwest Nigeria.

Cocoyam (*Xanthosoma sagittifolium* (L.) Schott) is an important tuber crop grown in many parts of the world, but a major staple food in Nigeria, South Pacific islands, and some part of Asia [18]. The corms and cormels are the major economic parts of cocoyam. Cocoyams are the cheapest and most handy source of carbohydrate in meals that are recommended for aged people, diabetics, convalescents, and most gastrointestinal disorder patients [19]. Moreso, Cocoyam, being a tuber crop, is sensitive to poor soil physical conditions [20] and potassium (K) in the soil [19]. Application of biochar could be a way of improving the physical and chemical soil condition and yield of cocoyam. Therefore, the objectives of this study were to determine the effects of biochar on soil physical and chemical properties, soil loss, and yield of cocoyam on a tropical Alfisol. In this experiment, we hypothesized that biochar will significantly (i) increase yield of cocoyam, (ii) improve soil physical and chemical properties, and (iii) reduce soil loss.

## 2. Materials and Methods

### 2.1. Experimental Site Treatments

Experiments were carried out at the Teaching and Research Farm, Rufus Giwa Polytechnic, Owo, Ondo State, Nigeria, in 2017 and 2018 seasons. The site lies between lat 7°12′N and long 5°35′E, 348 m above sea level, and is located in the forest-savanna transition zone of southwest Nigeria. The soil at Owo is an Alfisol classified as Oxic Tropludalf or Luvisol [21] derived from quartzite, gneiss, and schist [22]. The rainfall pattern is biomodal with peak in June and October. The total annual rainfall in the area is about 1350 mm while mean annual temperature is 32°C. The site of the experiment was just recovered from a fallow of five years after arable cropping to crops such as yam, cassava, melon, and cowpea for about one year without organic or inorganic fertilizer application.

The experiment each year consisted of four levels of biochar applied at 0, 10, 20, and 30 t ha^−1^. The four levels were laid out in a randomized complete block design (RCBD) and replicated three times. The plot size was 5 × 4 m. Blocks were 3 m apart while plots were 1 m apart. The same exact position and layout of plots and treatments were used for the experiment in 2017 and 2018.

### 2.2. Land Preparation, Incorporation of Biochar, and Planting of Cocoyam

Biochar was obtained from a local producer of charcoal that uses hardwood in a traditional kilns to produce charcoal for domestic use [4]. The temperature inside the kiln was monitored with a thermocouple and had an average temperature of 500°C for 12 hours. The pyrolysed biochar was later grounded and sieved with 2 mm sieve and made ready for application.

After manual clearing of the site of all weeds, ploughing was done in April each year to the depth of 20 cm. The site was layout to the required plot size of 5 × 4 m. The biochar was weighed and spread uniformly over the soil on plot basis to the required rates of 0, 10, 20, and 30 t ha^−1^ which was equivalent to 0, 20, 40, and 60 kg plot^−1^, respectively. Incorporation was done to the depth of 10 cm with a traditional hoe each year. The biochar was allowed two weeks before planting cocoyam.

Cocoyam (*Xanthosoma sagittifolium* cv. Owo local) cormels weighing about 150 g were planted [20]. One cormel of cocoyam was planted per hill at a spacing of 1 × 1 m to give a plant population of 20 plants per plot. Weeding was done manually at 45, 70, and 110 days after planting.

### 2.3. Determination of Soil Properties

In 2017 before the start of the experiment, soil samples from 0–0.15 m depth were randomly collected from 10 points from the experimental site. The soil samples were bulked together, air-dried, and sieved with 2 mm sieve for analysis (to serve as composite soil sample). The hydrometer was used for the determination of particle size [23]. Also, before the start of the experiment, bulk density of the experimental site was determined using the method of Campbell and Henshall [24]. Determination of soil chemical properties (SOM, N, P, K, Ca, Mg, and CEC) was also carried out by the procedure that had been fully described elsewhere [4, 22]. The air-dried soil samples that have passed through a 2 mm sieve were analysed for pH with pH meter and electrical conductivity (EC) with EC meter using 1 : 2 and 1 : 5 (soil: water) suspensions, respectively.

Two months after application of biochar, determination of selected soil physical properties on plots basis was started and was repeated on 2 other occasions in August and October each year. Core samplers (0.04 m diameter and 0.15 m high) in each occasion were used to collect soil sample from 0–0.15 m depth at about 0.1 m away from cocoyam plant. The soil samples were used to evaluate bulk density and gravimetric moisture content after oven drying at 100°C for 24 h. Soil porosity was calculated from the values of bulk density using the particle density value of 2.65 g cm^−3^.

Modified fast-wetting in water, as proposed by Bissonnais [25], was used to measure the aggregate stability of 2 mm air-dried aggregates (35 g). A 4 cm amplitude was applied for 5 min vertical movement to a nest of sieves (>2000, 1000–2000, 500–1000, 250–500, 250–106, <106 mm) immersed in a container of tap water (101 mS/cm). The material that remained after wet-shaking in each sieve was carefully removed, and the mean weight diameter (MWD) of the aggregate size was calculated using

(1) $$\begin{array}{c} \text{MWD}=\sum_{i=1}^{n}x_{i}w_{i}, \end{array}$$

where *n* is the number of sieves and *x* and *w* are diameter and weight, respectively.

Dispersion ratio was done by determining the amounts of silt and clay in calgon-dispersed as well as water-dispersed samples using the Bouyoucos hydrometer method of particle size analysis described in [23]. Dispersion ratio was determined as a measure of aggregate stability using the following formula:

(2) $$\begin{array}{c} \text{dispersion ratio}=\frac{\left(\%\text{silt}+\text{clay}\left({\text{H}}_{2}{\text{O}}_{2}\right)\right)}{\left(\text{\% silt}+\text{clay}\left(\text{calgon}\right)\right)}\times 100. \end{array}$$

Infiltration of water into the soil was determined in the experimental field using a double ring infiltrometer [26], with a 30 cm inner diameter and 60 cm outer diameter cylinder inserted 10 cm into the soil at the experimental plots. Water entering the soil was measured with a calibrated Marriott bottle. A constant water head of 20 mm was maintained in both rings [27].

At incorporation of biochar, five long (about 15 cm) nails adapted from Anikwe et al. [28] were randomly driven into the topsoil of each experimental plot perpendicular to the soil surface, and its exposure with time was used to monitor soil loss or soil removal by erosion from each plot. The length of each nail exposed in each plot was measured using a string and meter rule.

At the end of each year, soil samples were also collected on each experimental plot and analysed for soil chemical properties.

### 2.4. Determination of Cocoyam Yield

Ten plants were selected per plot for determination of cocoyam yield. Harvesting was done 7 months after planting. The cormel yield was determined by harvesting 10 cocoyam plants per plot removing the cormels from the corms. They were washed and cleaned to remove traces of sand before weighing on a top loading balance to determine their fresh weights.

### 2.5. Chemical Analysis of Biochar Used for the Experiment

About 5 g of the biochar and poultry manure used were collected and analysed for N, P, K, Ca, and Mg as described by Tel and Hagarty [29]. N was determined by the micro-Kjeldahl digestion method. The determination of P, K, Ca, and Mg was done using the wet digestion method based on 25-5-5 mL of HNO_3_-H_2_SO_4_-HClO_4_ acids. Phosphorus was measured colorimetrically by the molybdate blue method in an autoanalyser, K was measured by flame photometry, and Ca and Mg were measured by an atomic absorption spectrophotometer. Biochar was analysed for EC using 1 : 20 (biochar: water) suspension as described in [30].

### 2.6. Statistical Analysis

Data collected from each experiment were subjected to mean separation analysis using a two-way ANOVA test at a significance of *p*=0.05. The differences between mean values were identified using Duncan's multiple range test. Pearson's correlation coefficients were calculated to determine how the soil properties are related.

## 3. Results

### 3.1. Characteristics of Experimental Soil and Biochar Used

The soil of the experimental site (Table 1) was sandy loam with high bulk density, acidic, and low in soil nutrient except Mg [31]. The biochar used was alkaline (pH 7.6) in nature with high values of organic C, K, Ca Mg, C: N ratio and porosity compared with the preplanting soil (Table 1).

### 3.2. Response of Soil Physical Properties to Biochar Application

The responses of soil physical properties to biochar application are shown in Table 2. Application of biochar in both years reduced bulk density and increased porosity of the soil significantly compared with the control. Biochar reduced bulk density and increased porosity as the levels of the biochar increased with 30 t ha^−1^ biochar having the least bulk density and highest porosity. In the first year, 30 t ha^−1^ biochar reduced bulk density by 46.3% and increased porosity by 46.5% compared with no application of biochar. The reduction in bulk density was 74.7% and increases in porosity were 65.0% in the second year. Application of biochar at 10, 20, and 30 t ha^−1^ reduced bulk density and increased porosity by 4.3, 8.3, and 18.7%, respectively, in the second year compared with the first year. The interaction between year (*Y*) and biochar (*B*) (*Y* × *B*) for both bulk density and porosity was significant.

Soil moisture content, MWD, and infiltration rate increased significantly (Table 2) with the application of biochar compared with no application (control). These parameters significantly increased with the level of biochar. Year also increased moisture content, MWD, and infiltration rate significantly with 2018 having higher values. The interaction of *Y* × *B* was significant for moisture content, MWD, and infiltration rate.

Application of biochar reduced soil loss compared with the control. The highest soil loss (355.5 and 365.1 in 2017 and 2018, respectively) occurred in the control, and the lowest soil loss (118.0 and 68.25 in 2017 and 2018, respectively) occurred in the amended plots with the highest application rate 30 t ha^−1^ biochar. Soil loss was significantly reduced as the level of biochar increased. Using the mean of the two years, biochar at 30 t ha^−1^ reduced soil loss by 286.9% compared with the control. Second year (2018) significantly reduced soil loss compared with first year (2017). When 2018 is compared with 2017 in terms of soil loss, in the amended plots, 2018 reduced soil loss by 7.4, 20, and 73.5%, respectively, for 10, 20, and 30 t ha^−1^ biochar, whereas there was an increase of 2.7% soil loss in the control plot in 2018 compared with 2017. The interaction *Y* × *B* was significant for soil loss.

### 3.3. Response of Soil Chemical Properties to Biochar Application

Application of biochar increased soil chemical properties in the amended plots relative to the control in both years (Table 3), except pH and N in 2017. Also, in both years (except the case of no significant differences between 10 and 20 t ha^−1^ biochar levels for N, P, K, and Mg in 2017), biochar increased soil OM, N, P, K, Ca, Mg, and CEC from 0–30 t ha^−1^. There were no significant differences in the pH values between 20 and 30 t ha^−1^ biochar. The values of SOM, N, P, K, Ca, Mg, and CEC in 2018 were significantly higher than those of 2017. The interactive effect *Y* × *B* was significant for all soil chemical properties except pH.

### 3.4. Response of Cocoyam Yield to Biochar

**Figure 1:**
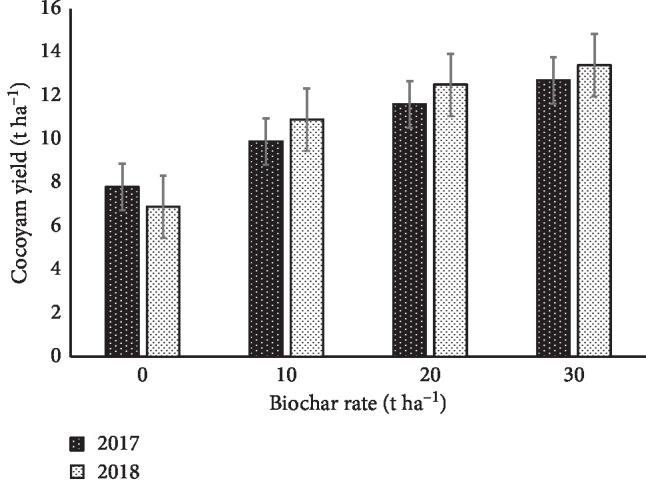
Effects of different rates of biochar on cocoyam yield in 2017 and 2018; vertical bars show standard errors.

Application of biochar increased the cormel yield of cocoyam significantly compared with the control (Figure 1). In both years, the yield of cocoyam was increased as the level of biochar increased from 0–30 t ha^−1^, and year 2018 increased yield of cocoyam compared with 2017. Compared with 2017, there was an increase in cocoyam yield by 8.1, 7.8, and 5.5% for 10, 20, and 30 t ha^−1^ biochar, respectively, and a reduction of 13% for the control. The interaction between *Y* × *B* was significant for cocoyam yield.

## 4. Discussion

The soil of the site of the experiment was low in nutrient, acidic, and fairly high in bulk density. These states of the soil are the characteristics of tropical soils [32, 33]. The fairly high bulk density of the site was partly related to its low organic matter content [34]. The reduced bulk density and increased porosity of the soil as a result of the application of biochar were due to the relatively lower bulk density of biochar relative to that of the soil. Also, biochar has high porosity (Table 1) which results from retaining the cell wall structure of the biomass feedstock [35]. Therefore, being a porous material when added to the soil, it increases its porosity and thus reduced bulk density [4, 36]. Hseu et al. [37] reported that the change in porosity with biochar-treated soils was as a result of formation of macropores and rearrangement of soil particle. Laird et al. [38] also reported similar finding and suggested that biochar is acting as a soil conditioner. The increase in porosity and decrease in bulk density as the level of biochar increased from 0–30 t ha^−1^ can be adduced to greater effects of biochar on porosity from each level of biochar application. This is in agreement with Kätterer et al.'s study [39] in Kenya where biochar addition increased soil porosity and water holding capacity after continuous addition for 10 years compared with bared soil. These results are also in agreement with those of Ndor et al. [40] where the applications of rice husk and sawdust biochars had a significant effect on soil moisture content, bulk density, porosity, and soil water-filled pore space.

In a 3-year field study [41], it was reported that biochar application reduced soil bulk density of 0–7.5 cm soil layer by 4.5 and 6.0% for 0.23 kg m^−2^ and 0.45 kg m^−2^ application rate, respectively.

Application of biochar increased moisture content of the soil compared with the control. This could be adduced to biochar soils having more micropores to physically retain water and or improved aggregation that resulted in creating more pore spaces as a result of greater earthworm burrowing. Another reason for the differences in water content between biochar-treated plots and the control could probably also be due to the differences in bulk density between treatments. The bulk density of the control plots was higher (reducing the spaces where water could be retained) compared with the bulk density of the biochar-treated plots [4]. Chan et al. [42] also reported that the water retention ability of biochar could be as a result of increase in overall net soil surface area in soil after biochar application. A long-term column study indicated that biochar amended Clarion soil retained up to 15% more water, and 13% and 10% more water retention at -100 kPa and -500 kPa soil matric potential respectively, compared with control [38]. The increase in moisture content with rates of biochar was adduced to increase in surface area for absorbing more moisture as the rates of biochar increase.

MWD increased significantly in biochar-amended soil compared with the control. MWD indicates prevalence of larger and more stable aggregates and therefore is an index of soil aggregate stability and quality [43, 44]. The increased MWD for plots with biochar could be due to increase in binding organic substances from the biochar, thereby improving the interparticular aggregate cohesion among the soil particles [45, 46]. Organic amendment has been known to enhance soil aggregate formation and stability [47]. The increase in soil aggregate stability following biochar application could be due to high carbon (C) associated with biochar [48]. The C molecules form bonds with the oxides, and the organic matter (OM) serves as food for soil microorganism making the environment favourable for them. The substrates supplied to the microorganisms by the labile OM on the surface of biochar enhance the excretion of mucilage by microorganism, which in turn builds stable soil aggregate [38]. There was a significant *Y* × *B* interaction for MWD; this can be related to increased organic C from the biochar which is an important binding agent in the formation and stability of soil aggregates [49, 50]. *Y* × *B* interaction could also be related to the active functional groups of biochar particles which may form complexes over time to make up soil aggregates [51].

Biochar-applied plots reduced dispersion ratio compared with the control. This was adduced to the OM from the biochar applied to soil. The biochar applied stabilized the soil structure and reduced dispersion ratio since organic matter addition is essential for stabilizing soil against physical degradation and soil erosion. Soils with high dispersion ratio are weak structurally and can easily be eroded [27].

The downward movement of water into the soil is known as infiltration. Biochar-applied plots increased infiltration rate compared with the control. This could be as a result of more pores created in the soil matrix as a result of biochar application because biochar is very porous. Prober et al. [52] reported an increase in water infiltration after a 2-year experiment in which biochar was applied at a rate of 20 Mg ha^−1^ to a clay loam soil.

The reduction in soil loss in the biochar plots compared with the control was adduced to increased aggregation which might have increased infiltration rate and therefore reduce runoff.  Table 4 shows that soil loss in this experiment was dependent among other factors: bulk density, porosity, moisture content, MWD, dispersion ratio, infiltration rate, and SOC. Application of biochar would have increased SOM which would have stabilized soil structure by increasing MWD, porosity, moisture content, and infiltration rate and reducing dispersion ratio and bulk density. Soils with high dispersion rate are weak structurally and can easily be eroded. Many researchers have used this index in predicting soil erosion by water [53]. Other researchers [6, 54] also showed that addition of biochar will increase SOM and therefore reduce soil loss by increasing the size of the soil aggregates as well as stabilizing soil aggregates. The reduced soil loss with increased rate of biochar was due to increased OM, better stabilization, and improved physical properties of the soil with the biochar rates. The improved soil physical properties and SOC in the second year were adduced to residual effect of biochar from the first season and subsequent application in the second year. This present result is in agreement with that of Agbede et al. [55] where incorporating biochar into the soil significantly reduced soil loss by 31%, 58%, and 82% at 10, 20, and 30 t ha^−1^ application rates, respectively, compared with the control on tropical Alfisol at Owo, southwest Nigeria.

Biochar improved soil chemical properties because of its ability to absorb soluble organic matter and inorganic nutrients [56]. Lehmann and Rondon [16] reported that biochar can adsorb both NH_4_^+^ and NH_3_^−^ from the soil solution. Biochar is very efficient at adsorbing dissolved solute nutrients such as ammonium [57], nitrate [58], phosphate [59], and other ionic solutes [60]. Biochar as reported by Jia et al. [61] can absorb leachate which can help to absorb organic matter, total soluble N, plant available P, and K, thereby increasing the nutrient retention capacity of the soil. The increase in K, Ca, Mg, and CEC in biochar-applied soils was due [62, 63] to the presence of cation exchange sites on the surface of biochar. Another reason for the high amount of cations in biochar soil may be due to the presence of carboxyl group in biochar which is indicated by high oxygen and carbon ratios on the surface of the biochar after microbial degradation [10, 64]. Soil chemical properties in 2018 were improved compared with 2017; this was due to the fact that biochar increased plant nutrient availability with age in the soil due to residual effect. The improved soil nutrients in 2018 compared with 2017 could also be as a result of the addition of new (fresh) biochar in 2018 which might induce net immobilization of inorganic N in already present in soil solution [65]. The increase in pH with biochar was due to the fact that biochar contains ash. These results of soil chemical properties with biochar are in agreement with the work of Njoku et al. [66] in which rice husk and sawdust biochar rates had a significant effect on all the chemical properties in the soil. 10 t ha^−1^ (the highest biochar rate) of rice husk and sawdust biochar produced the highest levels of pH, N, K, OC, Mg, Na, and CEC. This present result on soil chemical properties with biochar is also in agreement with that of Oguntunde et al. [67] in Ejura, Ghana, where there was a significant increase in soil pH, base saturation, exchangeable Ca, Mg, K, and Na, and available P in biochar-applied soils compared to the adjacent soils and that of Alling et al. [68] on tropical soils from Zambia and Indonesia where biochar has the ability to release essential plant growth nutrients as well as alleviate Al toxicity in those soils.

The increased yield of cocoyam in this study was due to improved physical and chemical characteristics of the soil as a result of biochar application. The improved soil physical properties favours reduced bulk density and increased porosity which would have enhanced better root penetration for nutrient absorption and also enhanced better tuberization of cocoyam cormels. Adekiya et al. [20] reported that cocoyam cormels are sensitive to high bulk density. Also, the enhanced availability of nutrient supply due to biochar in addition to soil physical properties also aided the yield of cocoyam. The increase in cations in biochar-amended plots brings an improvement in soil fertility and its nutrient retention [69] especially K that is important for tuber formation of cocoyam [19]. Adekiya et al. [4] reported that biochar alone because of its inert nature did not increase the yield of a short season crop like radish (*Raphanus sativus* L.) significantly in the first year of application; however, for cocoyam in this current study it did. This was due to the long period of growth of cocoyam (9 months) by which the biochar applied would have fully been oxidized and beneficial to the cocoyam. Singh et al. [70] reported that biochar develops reactive surfaces with time after exposure to water and oxygen in the soil. Major et al. [71] reported that the beneficial effect of applying biochar to soil improves with time. These improved soil chemical properties in the second year explain the yield differences of cocoyam between 2017 and 2018. The increase in cocoyam yield in this study agreed with Jeffery et al [73] that application of biochar may especially benefit crop production in low-nutrient, acidic soils in the tropics. This is in agreement with the result of Kätterer et al. [39] in Kenya where application of biochar had led to increases in the yields of maize and soya bean. Also, the result is in agreement with that of Njoku et al. [66] where application of sawdust and rice husk biochars at their highest rate of 10 t ha^−1^ produced the highest seed weight of sesame (*Sesamum indicum* L.) in Lafia, Nasarawa State, Nigeria. Reichenauer et al. [73] observed that the application of biochar, even at a very low dosage, would impact crop yield positively. Other crops such as maize [74], soybean [75], and upland rice [14] have been reported to increase in yield with application of biochar.

## 5. Conclusion

Field experimental results showed that biochar made from hardwood at a temperature of 500°C for 12 hours can be used to improve the yield of cocoyam and soil physical (bulk density, porosity, moisture content, mean weight diameter (MWD) of soil aggregates, dispersion ratio, and infiltration rate) and chemical (SOM, N, P, K, Ca, Mg, and CEC) properties and erosion resistance. These results suggest that the addition of wood biochar effectively improved poor soil characteristics in severely degraded sandy loam Alfisol and also reduced soil losses. The increased yield of cocoyam was due to improved soil physical and chemical properties. Therefore, biochar could be used to reduce rapid soil loss, improve soil quality, and increase cocoyam yield in tropical regions.

## Figures and Tables

**Table 1:** Properties of the experimental site and biochar prior to experimentation in 2017.

Properties	Soil	Biochar
Sand (%)	68.1 ± 1.3	NA
Silt (%)	16.2 ± 1.2	NA
Clay (%)	15.7 ± 1.1	NA
Textural class	Sandy loam	NA
Bulk density (Mg m^−3^)	1.57 ± 0.04	0.60 ± 0.03
Porosity (%)	40.75 ± 1.4	77.35 ± 1.5
Organic carbon (%)	1.17 ± 0.03	52 ± 1.2
Total N (%)	0.18 ± 0.01	0.65 ± 0.02
C : N ratio	6.5	80
Ash (%)	NA	0.49 ± 0.01
Available P (mg kg^−1^)	11.1 ± 0.3	0.36 ± 0.01
Exchangeable K (cmol kg^−1^)	0.10 ± 0.01	1.75 ± 0.02
Exchangeable Ca (cmol kg^−1^)	2.72 ± 0.03	4.51 ± 0.1
Exchangeable Mg (cmol kg^−1^)	0.42 ± 0.01	7.75 ± 0.1
pH (water)	5.69 ± 0.04	7.61 ± 0.05
Electrical conductivity (dS m^−1^)	0.11 ± 0.01	0.41 ± 0.02

**Table 2:** Effect of biochar on some selected soil physical properties in 2017 and 2018.

Year	Biochar rate (t ha^−1^)	Bulk density (Mg m^−3^)	Porosity (%)	Moisture content (%)	MWD (mm)	DR (%)	IR (cm hr^−1^)	Soil loss (cm)	Soil loss (kg ha^−1^)
2017	0	1.58a	40.4d	9.6d	1.06d	71a	10.6d	2.25a	355.5a
	10	1.44b	45.7c	11.2c	1.41c	65b	17.7c	1.75b	252.0b
	20	1.30c	50.9b	13.1b	1.61b	60c	21.4b	1.20c	156.0c
	30	1.08d	59.2a	14.9a	1.82a	55d	24.1a	1.10d	118.0d
2018	0	1.59a	40.0d	10.1d	1.05d	72a	10.1d	2.29a	365.1a
	10	1.38b	47.9c	12.6c	1.55c	61b	18.2c	1.70b	234.6b
	20	1.20c	54.7b	14.6b	1.71b	56c	23.4b	1.09c	130.8c
	30	0.91d	66.0a	15.9a	1.95a	48d	27.7a	0.75d	68.25d
Year (*Y*)	^*∗*^	^*∗*^	^*∗*^	^*∗*^	^*∗*^	^*∗*^	^*∗*^	^*∗*^	^*∗*^
Biochar (*B*)	^*∗*^	^*∗*^	^*∗*^	^*∗*^	^*∗*^	^*∗*^	^*∗*^	^*∗*^	^*∗*^
*Y* × *B*	^*∗*^	^*∗*^	^*∗*^	^*∗*^	^*∗*^	^*∗*^	^*∗*^	^*∗*^	^*∗*^

Values followed by similar letters under the same column are not significantly different at *p*=0.05 according to Duncan's multiple range test; MWD = mean weight diameter of soil aggregate; DR = dispersion ratio; IR = infiltration rate.

**Table 3:** Effect of biochar on soil chemical properties in 2017 and 2018.

Year	Biochar rate	pH (water)	OM (%)	N (%)	P (mg kg^−1^)	K (cmol kg^−1^)	Ca (cmol kg^−1^))	Mg (cmol kg^−1^))	CEC (cmol kg^−1^))
2017	0	5.61c	1.78d	0.16b	8.6c	0.09c	1.69d	0.35c	1.1d
	10	5.72bc	2.52c	0.17ab	10.1b	0.12b	1.72c	0.38b	3.4c
	20	5.88ab	2.75b	0.17ab	10.7b	0.13ab	1.92b	0.39b	5.3b
	30	5.96a	2.97a	0.18a	14.6a	0.14a	2.45a	0.44a	7.5a
2018	0	5.60d	1.70d	0.15d	7.1d	0.07d	1.61d	0.33d	1.0d
	10	5.83bc	2.70c	0.18c	12.6c	0.13c	1.84c	0.43c	5.6c
	20	6.10ab	2.89b	0.19bc	14.8b	0.15b	2.30b	0.56b	8.5b
	30	6.31a	3.18a	0.21a	17.7a	0.17a	2.79a	0.67a	12.9a
Year (*Y*)		ns	^*∗*^	^*∗*^	^*∗*^	^*∗*^	^*∗*^	^*∗*^	^*∗*^
Biochar (*B*)		^*∗*^	^*∗*^	^*∗*^	^*∗*^	^*∗*^	^*∗*^	^*∗*^	^*∗*^
*Y* × *B*		ns	^*∗*^	^*∗*^	^*∗*^	^*∗*^	^*∗*^	^*∗*^	^*∗*^

Values followed by similar letters under the same column are not significantly different at *p*=0.05 according to Duncan's multiple range test, ^*∗*^= significant at 5% level of probability; ns = not significant at 5% probability level.

**Table 4:** Correlation coefficient between soil properties.

	Bulk density	Porosity	Moisture content	MWD	DR	IR	Soil loss	SOM
Bulk density	1							
Porosity	−1.000^*∗∗*^	1						
Moisture content	−0.975^*∗∗*^	0.973^*∗∗*^	1					
MWD	−0.959^*∗∗*^	0.957^*∗∗*^	0.983^*∗∗*^	1				
DR	0.985^*∗∗*^	−0.984^*∗∗*^	−0.984^*∗∗*^	−0.985^*∗∗*^	1			
IR	−0.963^*∗∗*^	0.961^*∗∗*^	0.974^*∗∗*^	0.993^*∗∗*^	−0.983^*∗∗*^	1		
Soil loss	0.958^*∗∗*^	−0.956^*∗∗*^	−0.972^*∗∗*^	−0.979^*∗∗*^	0.973^*∗∗*^	−0.991^*∗∗*^	1	
SOM	−0.837^*∗∗*^	0.834^*∗*^	0.865^*∗∗*^	0.937^*∗∗*^	−0.897^*∗∗*^	0.931^*∗∗*^	−0.902^*∗∗*^	1

^*∗*^Significant difference at *p* = 0.05; ^*∗∗*^significant difference at *p*=0.01; MWD = mean weight diameter of soil aggregate; DR = dispersion ratio; IR = infiltration rate.

## Data Availability

All data used to support the findings of this study are included within the article.
